# Relationship between traditional and non-traditional obesity parameters and diabetes and early-onset diabetes: an analysis based on a large cohort

**DOI:** 10.3389/fendo.2025.1593917

**Published:** 2025-09-09

**Authors:** Miao Luo, Liying Huang, Huan Wang, Lei Zhao

**Affiliations:** ^1^ Department of Infectious Diseases, Union Hospital, Tongji Medical College, Huazhong University of Science and Technology, Wuhan, Hubei, China; ^2^ Department of Endocrinology, Union Hospital, Tongji Medical College, Huazhong University of Science and Technology, Wuhan, Hubei, China

**Keywords:** diabetes, early-onset diabetes, obesity, body mass index, prevention

## Abstract

**Background:**

Diabetes mellitus (DM), especially early-onset DM, poses a growing global health challenge. While body mass index (BMI) is commonly used to assess obesity, it does not adequately capture fat distribution or metabolic risk. Alternative indices such as waist-to-height ratio (WHtR) and conicity index (CI) may better predict diabetes risk, particularly in younger populations.

**Methods:**

This cohort study included 15,453 participants for overall DM risk analysis (mean follow-up: 6.04 years, range: 0.45–12.96) and 5,584 participants under age 40 for early-onset DM (mean follow-up: 3.38 years, range: 0.47–12.32). Ten obesity-related indices were evaluated. Cox regression models estimated the association between each index and the incidence of DM and early-onset DM. Time-dependent receiver operating characteristic (ROC) curves and C-index are used to assess discriminatory performance.

**Results:**

During follow-up, 373 cases of DM and 29 cases of early-onset DM were identified. WHtR showed the strongest association with DM risk (HR per SD=1.39; 95% CI: 1.24–1.56), while CI had the strongest association with early-onset DM (HR per SD=2.63; 95% CI: 1.88–3.66). The cardiovascular metabolic index (CMI) had the highest area under the ROC curve (AUC) for assessing short-term DM risk, while lipid accumulation products (LAP) had the highest AUC value for medium- to long-term DM risk. WHtR had the highest AUC of 0.80 in assessing the risk of early-onset DM.

**Conclusions:**

Several non-traditional obesity indices, particularly WHtR, CI, CMI, and LAP, are superior to BMI in assessing the risk of DM or early-onset DM. These indices may offer valuable tools for early detection and personalized prevention strategies in clinical practice.

## Introduction

Diabetes mellitus (DM) significantly threatens global health (1). Recent data shows that around 500 million people globally have DM, which is expected to rise to 1.3 billion by 2050 (2). Complications like cardiovascular disease, neuropathy, nephropathy, and retinopathy increase mortality and healthcare costs (3). DM is influenced by genetics, environment, lifestyle, and obesity (4). Obesity is a significant risk factor for DM due to its impact on insulin resistance, glucose tolerance, and hormone regulation (5). The global rise in obesity, caused by changes in diet and activity levels, highlights the importance of early identification and intervention for obese individuals (6).

Methods such as magnetic resonance quantification, dual-energy X-ray absorptiometry, and bioelectrical impedance analysis offer more accurate measurements of body fat content (7). However, these methods are unsuitable for large-scale populations due to high costs and the need for specialized equipment. Therefore, simple anthropometric indices are commonly employed. Body mass index (BMI) is currently the most widely used to classify obesity. However, the limitations of BMI have resulted in the emergence of the ‘‘obesity paradox,’’ where individuals with a high BMI have a lower risk of disease and death (8). To address this issue, waist circumference (WC) has been used to account for the neglected population with abdominal obesity (9). However, both BMI and WC fail to reflect an individual’s obesity status accurately. Several novel obesity indices have been proposed (10–17), including waist-to-height ratio (WHtR), cardiometabolic index (CMI), lipid accumulation product (LAP), visceral adiposity index (VAI), a body shape index (ABSI), body roundness index (BRI), conicity index (CI), and atherogenic index of plasma (AIP). Studies have been conducted to explore the relationship between these traditional and non-traditional obesity indices and DM (10, 12–15). However, it remains unclear which obesity indices are most strongly associated with DM and which are the most reliable predictors of short-, medium-, and long-term DM risk. Additionally, early-onset DM (onset before the age of 40) is of particular concern due to its worse metabolic status and higher risk of complications (18). As the trend towards younger age of DM continues, the number of patients with early-onset DM is rapidly increasing (19). Existing research suggests that early-onset DM is more closely linked to obesity (20). Nevertheless, there is a significant lack of research on the occurrence of early-onset DM, and the relationship between the indices above and early-onset DM remains unclear.

Therefore, to fill the gap in the existing literature on these issues, we investigate the relationship between different obesity indices and the risk of DM and early-onset DM in the general population.

## Methods

### Study population

The population in the study conducted at Murakami Memorial Hospital in Gifu, Japan, consisted of individuals who underwent a physical examination and were part of the NAGALA (NAfld in the Gifu Area, Longitudinal Analysis) database. The original study (21) established a DM risk cohort by excluding participants who had used any medication at the beginning of the study, had been previously diagnosed with DM, or had a fasting glucose level of ≥ 6.1 mmol/L. For the present study, the analysis was conducted on this cohort, and an additional risk cohort of early-onset DM was identified by excluding individuals aged ≥40 years (**Supplementary Figure 1**).

### Individual characteristics collection

Medication history and lifestyle information, including smoking, alcohol consumption, and physical activity, were gathered using a standardized self-administered questionnaire. Age, gender, height, weight, blood pressure, and blood parameters were acquired from hospital-based physical examination data. Further elaboration can be found in a previously published article (21).

### Follow-up and outcome definitions

All participants in the study underwent a minimum of two physical examinations at the institution, with over 60% receiving 1–2 physical examinations annually. The diagnosis of DM was based on the criteria set by the World Health Organization, which included a fasting plasma glucose (FPG) level of 7.0 mmol/L or higher, a hemoglobin A1c (HbA1c) level of 6.5% or higher, or self-reported DM. Early-onset DM was defined as the onset of DM before the age of 40, and if the age at the last follow-up exceeded 40 years, the follow-up duration was calculated as “40 minus age”. The level of insulin resistance was assessed using the metabolic score for insulin resistance (METS-IR) and was calculated as (22):


$$\begin{array}{c} METS-IR=L\text{n}[2\times FPG(mg/dL)+TG(mg/dL)]\\ \times BMI(kg/m^{2})/Ln[HDL-C(mg/dL)] \end{array}$$

### Definition of obesity-related indices

Each obesity index is calculated as follows, Equations 1–9 respectively:


(1) $$BMI=Weight(kg)/{\left[Height(m)\right]}^{2}$$

(2) WHtR=WC(cm)/Height(cm)

(3) $$\begin{array}{l} VAI(males)=\frac{WC(cm)}{39.68+1.88\times BMI}\times \frac{TG(mmol/L)}{1.03}\times \frac{1.31}{HDL(mmol/L)}\\ VAI(females)=\frac{WC(cm)}{36.58+1.89\times BMI}\times \frac{TG(mmol/L)}{0.81}\times \frac{1.52}{HDL(mmol/L)} \end{array}$$

(4) LAP=TG(mmol/L)×[WC(cm)−(65, if males; 58 if females)]

(5) $$CI=\frac{WC(\text{m})}{0.109\times \sqrt{W\text{eig}ht(kg)/Height(m)}}$$

(6) $$BRI=364.2-365.5\times \sqrt{1-{\left[\frac{WC(cm)/2\pi ]}{0.5\times Height(cm)}\right]}^{2}}$$

(7) $$ABSI=\frac{WC(cm)}{{\left[Height(cm)\right]}^{1/2}\times BMI^{2/3}}$$

(8) $$CMI=\frac{TG(mmol/L)}{HDL-C(mmol/L)}\times WHtR$$

(9) AIP=log[TG(mmol/L)/HDL(mmol/L)]

### Statistical analysis

The DM and early-onset DM risk cohorts were initially separated based on when DM occurred. Baseline characteristics of both groups were compared using t-tests or Mann-Whitney U tests based on normality test results. Categorical variables were presented as n (%) and analyzed using a chi-square test, with Fisher’s exact test used for counts less than 10. Obesity measures were grouped into quartiles (**Supplementary Table 1**), and a trend analysis compared their relationship with DM and early-onset DM. Lastly, the Spearman correlation coefficient was employed to assess the association between obesity indices and METS-IR.

To address differences in units, we standardized obesity indices with z-scores. We used multivariable Cox regression to examine the relationship between obesity indices and the development of DM and early-onset DM, providing insights into changes in obesity indices per SD increase. Model 1 was adjusted for age and gender, while model 2 was additionally adjusted for systolic blood pressure (SBP), FPG, HbA1c, lipid levels (total cholesterol [TC], triglyceride [TG], high-density lipoprotein cholesterol [HDL-C]), liver enzyme levels (alanine aminotransferase [ALT], aspartate aminotransferase [AST], gamma-glutamyl transferase [GGT]), lifestyle habits (smoking, alcohol consumption, and exercise), and fatty liver. The relationships between covariates were evaluated using variance inflation factors (VIF), with a VIF value greater than 5 indicating collinearity. Based on Model 2, RCS plots were employed to examine potential nonlinear associations between obesity indices and outcome events. Models with 3–7 knots (**Supplementary Table 2**) were assessed individually, and the model with the lowest Akaike Information Criterion (AIC) was chosen to construct the restricted cubic spline. In cases where a nonlinear relationship was present, recursion was utilized to identify the inflection point, and segmented multivariable Cox regressions were conducted based on this inflection point. Due to the limited number of cases (29) in the early-onset DM risk cohort outcome event, the multivariable analysis procedure solely accounted for age and gender, adhering to the ten events per variable (EPV) criterion.

Sensitivity analyses were done to check the reliability of the results. Individuals with a two-year follow-up were excluded to prevent reverse causality. The e-value was calculated to measure unmeasured confounding in the relationship between obesity and the outcome. Stratified analyses were conducted in different groups based on age, gender, fatty liver, and hypertension levels, with interactions examined to assess the consistency of results among various populations.

Finally, we used time-dependent ROC curves to evaluate how well different obesity indices predicted the onset of DM over 2 to 12 years. The cohort with early-onset DM relied on ROC curves for the 12-year outcome, with Harrell’s concordance index (C-index) used for consistency testing. All analyses were conducted using R software (version 4.2.1), with a two-sided p-value < 0.05 considered statistically significant.

## Results

### Baseline characteristics

The study included a DM risk cohort consisting of 15,453 individuals with a mean age of 43.71 (8.90) years. Over a mean follow-up period of 6.04 years (0.45-12.96 years), DM was diagnosed in 373 individuals (399 per 100,000 person-years). Additionally, there was an early-onset DM risk cohort comprising 5584 individuals with a mean age of 34.82 (3.55) years. During a mean follow-up period of 3.38 years (0.47-12.32 years), 29 individuals (154 per 100,000 person-years) developed DM.

**Table 1** shows baseline characteristics of study population, grouped by DM occurrence. All parameters showed significant differences between DM and non-DM groups (*P* < 0.05). Those who developed DM had higher FPG, HbA1c, and METS-IR values (standardized mean difference, SMD >1) than non-DM individuals. There were also notable differences in ALT, TG, HDL-C, and fatty liver prevalence (SMD: 0.67-0.99). All obesity indices, except ABSI, were higher in the group that developed DM. The cohort with early-onset DM had significant differences in baseline characteristics, excluding age, height, and lifestyle factors. Participants who developed DM during the study had higher levels of ALT, FPG, HbA1c, METS-IR, fatty liver prevalence, and lower levels of HDL-C, similar to those in the DM group. Obesity indices, except ABSI, also showed significant differences compared to those without DM.

**Table 1:** Baseline characteristics of participants.

Characteristics	DM		*P* value	SMD (95% CI)	Early-onset DM		*P* value	SMD (95% CI)
	No	Yes			No	Yes		
*N*	15080	373			5555	29		
Age, years	43.63 (8.89)	47.14 (8.52)	<0.001	**0.40 (0.30, 0.51)**	34.82 (3.55)	34.76 (2.90)	0.495	0.02 (-0.35, 0.38)
**Gender**			<0.001	**0.49 (0.39, 0.59)**			0.017	**0.48 (0.11, 0.84)**
Females	6947 (46.07%)	87 (23.32%)			2567 (46.21%)	7 (24.14%)		
Males	8133 (53.93%)	286 (76.68%)			2988 (53.79%)	22 (75.86%)		
Height, m	1.65 (0.08)	1.67 (0.09)	<0.001	**0.19 (0.09, 0.29)**	1.66 (0.08)	1.69 (0.08)	0.106	**0.28 (-0.08, 0.65)**
Weight, kg	60.41 (11.48)	69.84 (13.32)	<0.001	**0.76 (0.66, 0.86)**	60.64 (12.43)	75.79 (17.15)	<0.001	**1.01 (0.65, 1.38)**
SBP, mmHg	114.31 (14.91)	122.03 (15.59)	<0.001	**0.51 (0.40, 0.61)**	111.90 (13.70)	120.43 (13.31)	<0.001	**0.63 (0.27, 1.00)**
DBP, mmHg	71.44 (10.47)	77.18 (10.23)	<0.001	**0.55 (0.45, 0.66)**	69.04 (9.60)	75.09 (9.87)	0.001	**0.62 (0.26, 0.99)**
ALT, U/L	17.00 (10.00)	24.00 (21.00)	<0.001	**0.67 (0.56, 0.77)**	16.00 (11.00)	32.00 (38.00)	<0.001	**0.81 (0.45, 1.18)**
AST, U/L	17.00 (10.00)	20.00 (10.00)	<0.001	**0.44 (0.34, 0.55)**	17.00 (7.00)	21.00 (15.00)	<0.001	**0.59 (0.22, 0.95)**
GGT, U/L	15.00 (11.00)	24.00 (19.00)	<0.001	**0.47 (0.37, 0.58)**	14.00 (9.00)	25.00 (25.00)	<0.001	**0.63 (0.27, 1.00)**
TC, mmol/L	5.12 (0.86)	5.43 (0.90)	<0.001	**0.35 (0.25, 0.46)**	4.85 (0.81)	5.16 (0.94)	0.046	**0.35 (-0.01, 0.72)**
TG, mmol/L	0.72 (0.62)	1.21 (1.07)	<0.001	**0.73 (0.62, 0.83)**	0.62 (0.54)	0.94 (0.96)	<0.001	**0.60 (0.24, 0.97)**
HDL-C, mmol/L	1.47 (0.40)	1.19 (0.33)	<0.001	**0.77 (0.66, 0.87)**	1.47 (0.39)	1.16 (0.38)	<0.001	**0.81 (0.44, 1.17)**
FPG, mmol/L	5.15 (0.41)	5.61 (0.36)	<0.001	**1.21 (1.11, 1.32)**	5.09 (0.40)	5.59 (0.44)	<0.001	**1.19 (0.82, 1.55)**
HbA1c, %	5.16 (0.32)	5.53 (0.37)	<0.001	**1.07 (0.97, 1.18)**	5.11 (0.29)	5.56 (0.33)	<0.001	**1.43 (1.06, 1.80)**
METS-IR	30.98 (6.36)	38.58 (7.74)	<0.001	**1.07 (0.97, 1.18)**	30.36 (6.72)	40.75 (9.41)	<0.001	**1.27 (0.91, 1.64)**
**Obesity-related indices**								
BMI	22.04 (3.07)	25.03 (3.82)	<0.001	**0.86 (0.76, 0.97)**	21.77 (3.31)	26.45 (4.89)	<0.001	**1.12 (0.75, 1.48)**
WC	76.25 (8.97)	85.08 (10.20)	<0.001	**0.92 (0.82, 1.02)**	75.01 (9.18)	88.15 (14.48)	<0.001	**1.08 (0.72, 1.45)**
WHtR	0.46 (0.05)	0.51 (0.06)	<0.001	**0.90 (0.80, 1.00)**	0.45 (0.05)	0.52 (0.08)	<0.001	**1.06 (0.70, 1.43)**
VAI	0.72 (0.77)	1.52 (1.59)	<0.001	**0.74 (0.64, 0.84)**	0.61 (0.66)	1.44 (1.12)	<0.001	**0.66 (0.29, 1.02)**
LAP	9.52 (14.28)	26.89 (32.67)	<0.001	**0.86 (0.76, 0.96)**	7.04 (11.35)	24.39 (31.05)	<0.001	**0.87 (0.51, 1.24)**
CI	1.16 (0.07)	1.21 (0.07)	<0.001	**0.72 (0.61, 0.82)**	1.14 (0.06)	1.21 (0.09)	<0.001	**0.86 (0.50, 1.23)**
CMI	0.23 (0.29)	0.58 (0.70)	<0.001	**0.78 (0.68, 0.89)**	0.19 (0.25)	0.55 (0.54)	<0.001	**0.73 (0.37, 1.10)**
AIP	-0.69 (-1.09)	0.09 (-1.10)	<0.001	**0.92 (0.81, 1.02)**	-0.86 (-1.08)	-0.08 (-0.37)	<0.001	**0.74 (0.38, 1.11)**
ABSI	0.76 (0.04)	0.77 (0.04)	<0.001	**0.40 (0.30, 0.50)**	0.75 (0.04)	0.76 (0.04)	0.013	**0.47 (0.10, 0.83)**
BRI	2.72 (0.88)	3.63 (1.18)	<0.001	**0.88 (0.78, 0.98)**	2.53 (0.86)	3.91 (1.78)	<0.001	**0.99 (0.63, 1.36)**
Fatty liver	2514 (16.67%)	223 (59.79%)	<0.001	**0.99 (0.89, 1.09)**	797 (14.35%)	19 (65.52%)	<0.001	**1.23 (0.86, 1.59)**
Regular exercise	2655 (17.61%)	51 (13.67%)	0.048	**0.11 (0.01, 0.21)**	781 (14.06%)	2 (6.90%)	0.268	**0.24 (-0.13, 0.60)**
**Smoking**			<0.001	**0.45 (0.35, 0.55)**			0.267	**0.28 (-0.08, 0.65)**
Never	8882 (58.90%)	145 (38.87%)			3444 (62.00%)	15 (51.72%)		
Ever	2872 (19.05%)	77 (20.64%)			892 (16.06%)	4 (13.79%)		
Current	3326 (22.06%)	151 (40.48%)			1219 (21.94%)	10 (34.48%)		
**Drinking**			<0.001	**0.21 (0.11, 0.31)**			0.163	**0.37 (0.01, 0.74)**
None/small	11536 (76.50%)	266 (71.31%)			4544 (81.80%)	23 (79.31%)		
Light	1714 (11.37%)	40 (10.72%)			551 (9.92%)	1 (3.45%)		
Moderate	1320 (8.75%)	37 (9.92%)			342 (6.16%)	3 (10.34%)		
Heavy	510 (3.38%)	30 (8.04%)			118 (2.12%)	2 (6.90%)		

Data are expressed as mean (SD), median (IQR), and n (%). Bolded values indicate statistically significant differences.

DM, diabetes mellitus; SMD, standardized mean difference; SBP, systolic blood pressure; DBP, diastolic blood pressure; ALT, alanine aminotransferase; AST, aspartate aminotransferase; GGT, gamma-glutamyl transferase; TC, total cholesterol; TG, triglyceride; FPG, fasting plasma glucose; HDL-C, high-density lipoprotein cholesterol; HbA1c, hemoglobin A1c; METS-IR, metabolic score for insulin resistance; BMI, body mass index; WC, waist circumference; WHtR, waist-to-height ratio; CMI, cardiometabolic index; LAP, lipid accumulation product; VAI, visceral adiposity index; ABSI, a body shape index; BRI, body roundness index; CI, conicity index; AIP, atherogenic index of plasma.

### Correlation analysis of obesity indices

Spearman correlation test revealed that all obesity indices were positively correlated with each other (all r >0 and *P <*0.05, **Supplementary Figure 2**). Within the DM risk cohort, WHtR exhibited the strongest correlation with BRI (r=1), followed by CMI and AIP (r=0.99), VAI (r=0.97), and ABSI displayed a significant correlation with CI (r=0.91). The weakest correlation was observed between BMI and ABSI (r=0.09). These findings were consistent in the early-onset DM risk cohort as well. Except for CI and ABSI, the remaining obesity indices were strongly correlated with METS-IR (all r > 0.7, **Supplementary Table 3**).

### Relationship between obesity-related indices and DM

**Figure 1:**
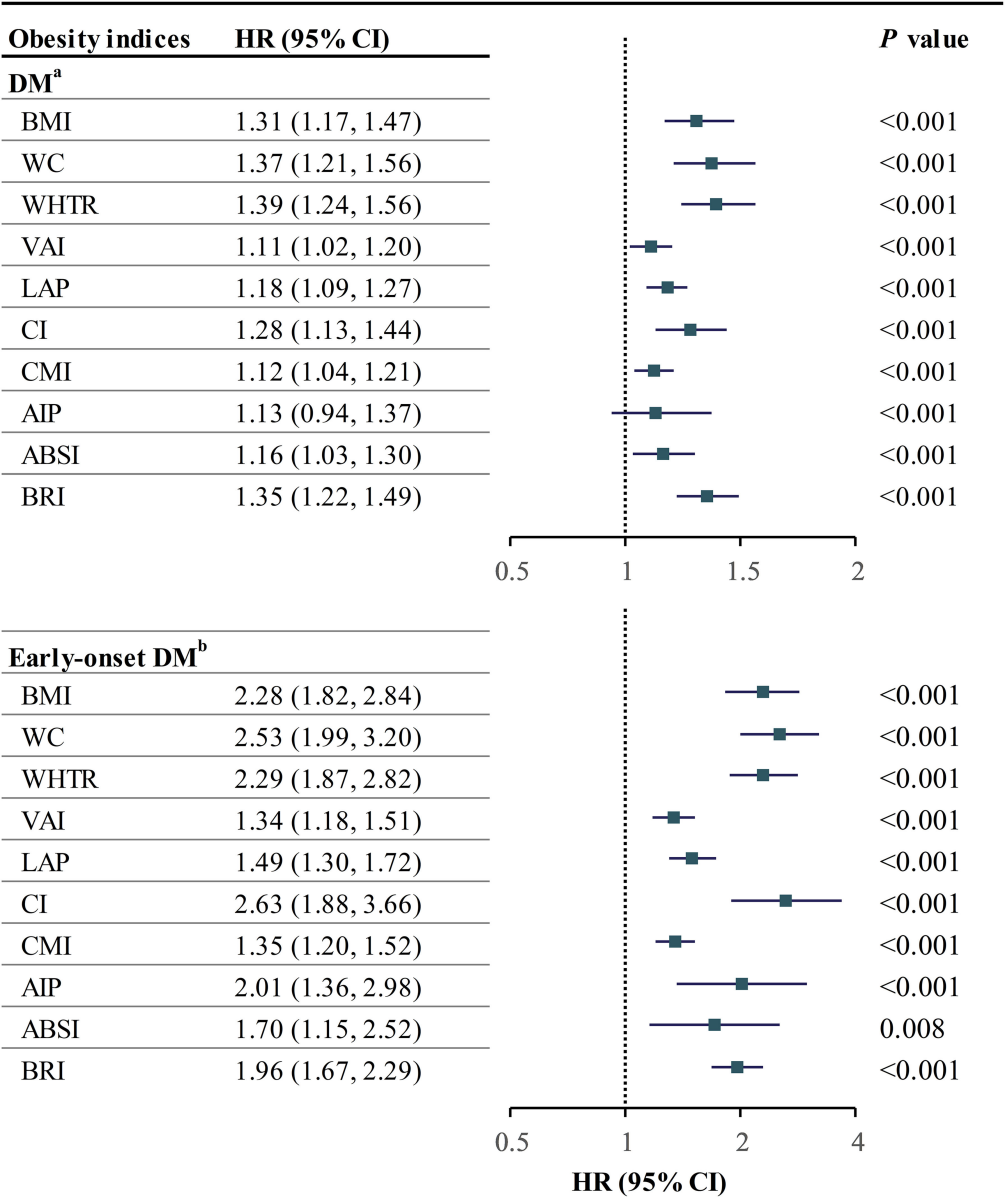
Forestplot of obesity indices with DM and early-onset DM. ^a^adjusted for age, gender, SBP, FPG, HbA1c, TC, TG, HDL, ALT, AST, GGT, smoking, drinking, and exercise; ^b^adjusted for age and gender; HR hazard ratio, CI confidence interval, other abbreviations can be seen in **Table 1**.

Our results showed that individuals with the highest obesity index had the highest rate of DM (**Supplementary Figure 3**). We used Cox regression models to analyze the relationship between obesity indices and DM, following the strengthening of the reporting of observational studies in epidemiology (STROBE) guidelines. Models included unadjusted, minimum-adjusted, and maximum-adjusted variables (23). (**Supplementary Table 6**). We tested the validity of our results using a proportional risk hypothesis test and found no violations of the assumption (**Supplementary Table 4**). None of the covariates showed collinearity. In Model 2, all obesity indices were positively correlated with the risk of DM, with statistically significant differences observed except for AIP (**Supplementary Table 5**). Notably, WHtR (per SD) demonstrated the strongest association with the risk of DM, as evidenced by a hazard ratio of 1.39 (95% CI: 1.24-1.56, **Figure 1**).

After adjusting for all covariates, it was found that WC, WHtR, VAI, CI, and BRI have nonlinear relationships with the risk of developing DM (**Supplementary Figure 5**). Recursive analysis was used to determine the inflection points (**Supplementary Table 7**). WC had a negative association with DM at 72.1-78.5 cm and a positive association above 78.5 cm. VAI had a U-shaped relationship with DM at a cutoff point of 0.43. WHtR, CI, and BRI were positively associated with DM beyond the second inflection point.

### Relationship between obesity-related indices and early-onset DM

Similar to the findings in the DM risk cohort, individuals with an obesity index in the Q4 quartile exhibited the highest prevalence of early-onset DM (**Supplementary Figure 4**). Given the limited occurrence of early-onset DM cases, only unadjusted and minimally adjusted (Model 1) Cox regression models were developed per the 10EPV rule. All measures of obesity showed a higher risk of early-onset DM, with CI having the strongest association. BMI, VAI, LAP, and CMI had a nonlinear relationship with early-onset DM. Limited events prevented further analysis.

### Sensitivity analysis

In the DM risk cohort, the association between obesity index and DM remained consistent after adjusting for covariates and excluding individuals with less than two years of follow-up (**Supplementary Table 6**). These e-values in **Supplementary Table 8** indicate the minimum relative risk ratio (RR, 1.46-2.13) required for unmeasured confounders to alter the current outcome. Finally, we analyzed the relationship between obesity indices and the presence of DM within different age groups (20-39, 40-59, ≥60), genders (male and female), and subgroups based on the presence or absence of fatty liver and hypertension. Our findings indicate that WC, WHtR, and CI are positively associated with DM solely within the population with fatty liver (*P*_interaction_ < 0.05, **Supplementary Table 9**). Conversely, BMI, WHtR, and BRI did not demonstrate statistically significant associations with DM within the hypertensive population. The remaining obesity indices yielded consistent results across all people.

In the cohort of individuals with early-onset DM, the impact of unmeasured confounding on the outcome was assessed solely through the computation of the e-value, given the limited occurrence of events. **Supplementary Table 8** reveals that a minimum RR of 2.01-4.7 is necessary for unmeasured confounders to reverse the association between the obesity index (as per Model 1) and early-onset DM.

### Predictive value of obesity indices

**Figure 2:**
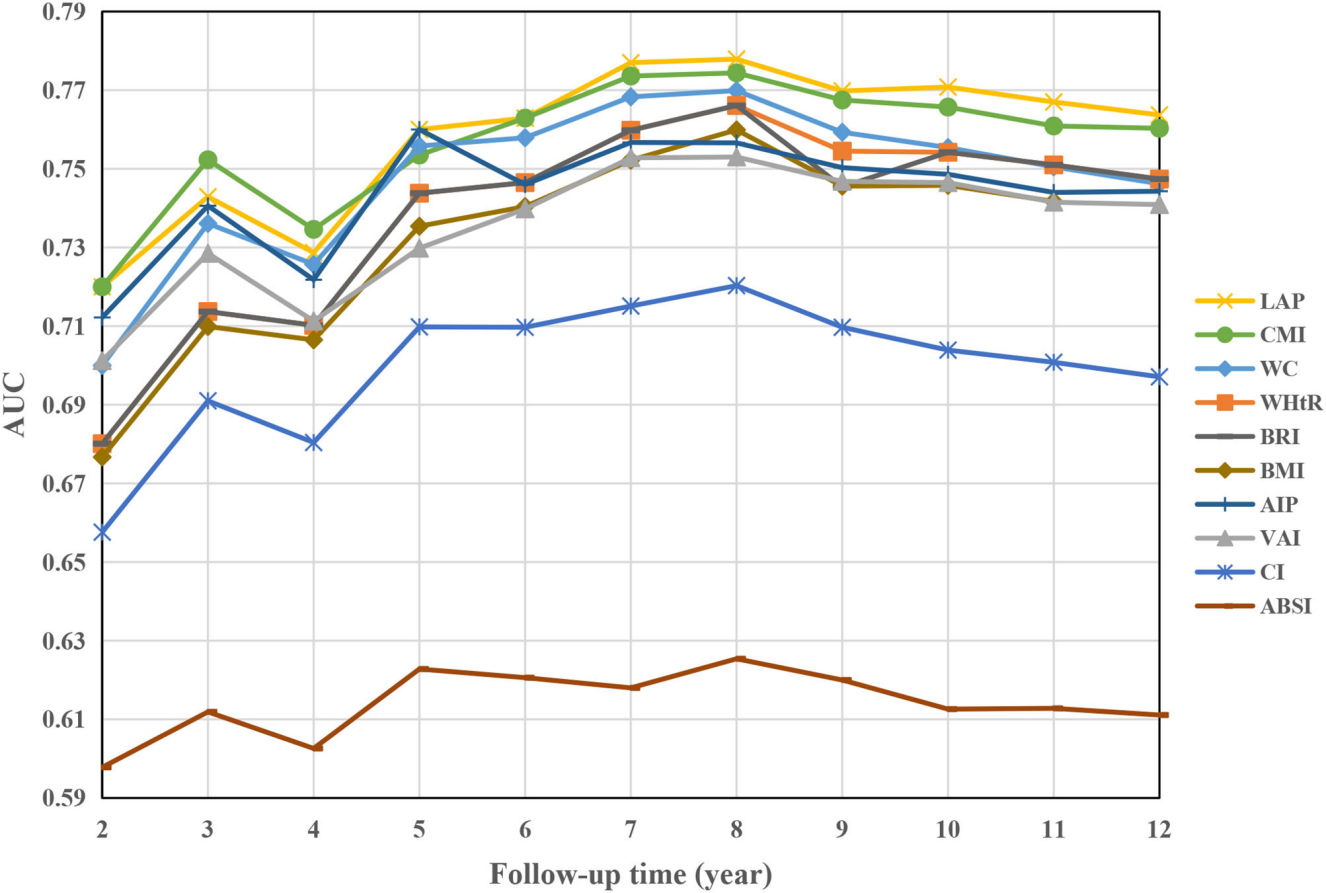
AUC of obesity indices over time to predict future DM risk. AUC area under the curve, other abbreviations can be seen in **Table 1**.

**Figure 2** illustrates the variations in the area under the curve (AUC) of each obesity index in predicting the development of DM over 2–12 years. The AUC values were highest for predicting short-term (2–4 years) DM risk using the CMI index, while the LAP index demonstrated the highest AUC values for predicting medium- to long-term (4–12 years) DM risk. Notably, the optimal cutoff values, calculated from the Youden index, remained relatively stable for each index, except for the AIP index (**Table 2**). Furthermore, the LAP index exhibited the highest C-index (0.757, 95% CI: 0.730-0.784, **Supplementary Table 10**). According to pairwise Hanley & McNeil test comparisons (**Supplementary Table 11**), BMI was not statistically inferior to WC, WHtR, VAI, AIP, or BRI in terms of discrimination. However, CMI and LAP showed significantly better discrimination than BMI (*P*=0.035 and 0.002, respectively).

**Table 2:** Best thresholds and AUCs for each obesity index to predict future DM risk.

Predict time	Incidents	AUC (Best threshold)									
		BMI	WC	WHtR	VAI	LAP	CI	CMI	AIP	ABSI	BRI
2-year	61	0.68 (21.23)	0.70 (81.95)	0.68 (0.48)	0.70 (1.63)	0.72 (14.32)	0.66 (1.16)	**0.72 (0.48)**	0.71 (0.11)	0.60 (0.75)	0.68 (3.08)
3-year	101	0.71 (24.91)	0.74 (83.25)	0.71 (0.48)	0.73 (1.06)	0.74 (14.32)	0.69 (1.20)	**0.75 (0.44)**	0.74 (0.11)	0.61 (0.76)	0.71 (3.07)
4-year	127	0.71 (25.05)	0.73 (81.95)	0.71 (0.48)	0.71 (1.09)	**0.73 (16.64)**	0.68 (1.19)	**0.73 (0.31)**	0.72 (0.11)	0.60 (0.76)	0.71 (6.06)
5-year	161	0.74 (25.05)	0.76 (84.45)	0.74 (0.5)	0.73 (1.05)	**0.76 (16.64)**	0.71 (1.20)	0.75 (0.31)	**0.76 (-0.45)**	0.62 (0.75)	0.74 (3.36)
6-year	192	0.74 (23.55)	0.76 (84.75)	0.75 (0.5)	0.74 (1.09)	**0.76 (16.64)**	0.71 (1.19)	**0.76 (0.31)**	0.75 (-0.45)	0.62 (0.75)	0.75 (3.29)
7-year	234	0.75 (23.55)	0.77 (80.75)	0.76 (0.5)	0.75 (1.05)	**0.78 (16.64)**	0.72 (1.19)	0.77 (0.31)	0.76 (-0.37)	0.62 (0.75)	0.76 (3.29)
8-year	262	0.76 (23.55)	0.77 (83.15)	0.77 (0.5)	0.75 (1.05)	**0.78 (16.64)**	0.72 (1.19)	0.77 (0.31)	0.76 (-0.37)	0.63 (0.75)	0.77 (3.29)
9-year	288	0.75 (23.53)	0.76 (83.15)	0.75 (0.5)	0.75 (1.05)	**0.77 (16.57)**	0.71 (1.19)	**0.77 (0.31)**	0.75 (-0.45)	0.62 (0.75)	0.75 (3.29)
10-year	318	0.75 (23.53)	0.76 (83.15)	0.75 (0.5)	0.75 (1.05)	**0.77 (16.14)**	0.7 (1.19)	**0.77 (0.31)**	0.75 (-0.37)	0.61 (0.75)	0.75 (3.29)
11-year	347	0.74 (23.53)	0.75 (81.05)	0.75 (0.5)	0.74 (1.05)	**0.77 (16.05)**	0.7 (1.19)	0.76 (0.34)	0.74 (-0.29)	0.61 (0.76)	0.75 (3.29)
12-year	364	0.74 (23.53)	0.75 (81.05)	0.75 (0.5)	0.74 (1.05)	**0.76 (16.05)**	0.7 (1.19)	**0.76 (0.34)**	0.74 (-0.29)	0.61 (0.76)	0.75 (3.29)

AUC, area under the curve, other abbreviations can be seen in **Table 1**. Bolded values represent the largest AUC values among all obesity indices.

**Figure 3:**
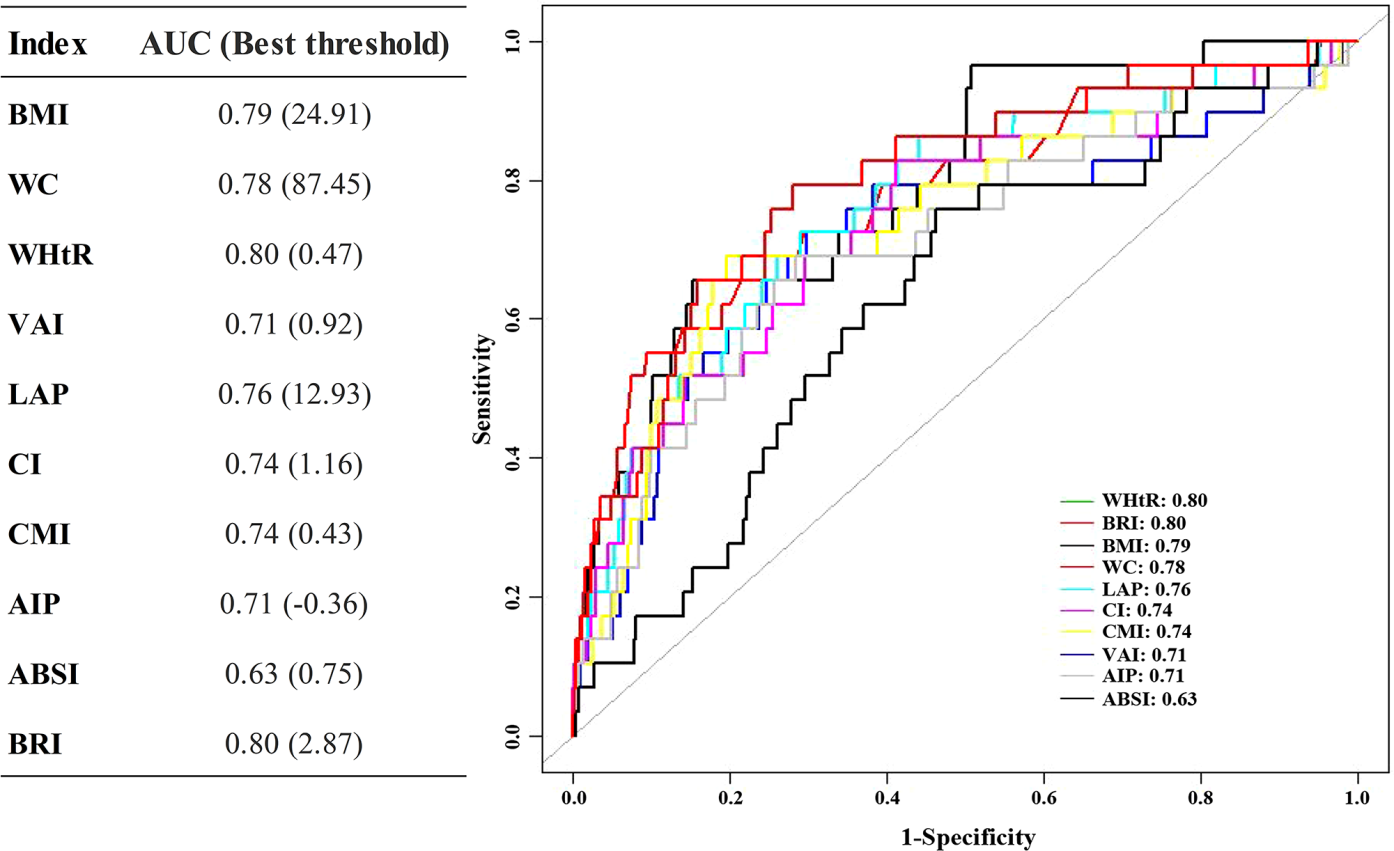
ROC of obesity indices predicts the 12-year DM risk. ROC receiver operating characteristic, other abbreviations can be seen in **Table 1**.

The calculation of early-onset DM was limited to assessing the effectiveness of each obesity index in predicting outcomes over 12 years, as there were fewer events available for analysis (**Figure 3**). Among the obesity indices considered, WHtR demonstrated the highest AUC value of 0.80 (95% CI: 0.71-0.88) and C-index of 0.772 (95% CI: 0.664-0.880).

## Discussion

This study found that various obesity-related indices are positively linked to the development of DM and early-onset DM, supporting the idea that obesity is a major factor in these conditions. Although non-traditional indices such as WHtR and CI showed stronger associations, BMI demonstrated comparable predictive ability and remains a practical, efficient tool for DM risk assessment.

WHtR showed the strongest link to DM, followed by WC and BRI. WHtR is a better indicator of abdominal obesity (24), which is strongly linked to DM. WHtR and WC are more closely tied to DM risk than BMI (24, 25). Additionally, BRI, which WC and height also determine, showed a perfect correlation with WHtR (spearman r=1, *P* < 0.001), consistent with a previous study (26). However, similar to previous studies (27), the results of this study also indicate that BRI is not as strongly related to DM as WHtR and WC. The main reason may be that BRI can reflect visceral and total body fat content (26). In contrast, the present study population may have more subcutaneous fat (28), thus attenuating the effect of BRI on DM. CMI incorporates additional risk factors for DM, such as TG and HDL-C, WC, and height. However, the association with DM is not as strong as the aforementioned indices, which have been reported in previous studies (29). Based on the available literature, it is hypothesized that this weaker association may be due to WHtR and TG/HDL not increasing in parallel. For instance, non-obese patients with fatty liver disease may have lipid abnormalities (30), but their BMI and WC remain within normal range. Therefore, the combined effect of WHtR and TG/HDL on DM is weakened. Similarly, VAI and LAP, which are defined similarly to CMI, may also perform poorly for this reason (11, 12).

Changes in TG and HDL-C are key indicators of insulin resistance. The atherogenic index of plasma (AIP), made up of TG and HDL, can detect hidden fat (17) and is linked to cardiovascular disease and DM (31). Obesity leading to DM has multiple causes (5). AIP does not consider other factors like free fatty acids and LDL-C on DM, so its impact on DM is reduced after adjusting for factors like TC. A study in Japan found that obesity has minimal influence on lipid levels (32). Another index, ABSI, independent of BMI and WC, has shown better associations with cardiovascular disease and all-cause mortality than these indices (33). ABSI has a weaker link to DM compared to BMI and WC (34). A study by Chang et al. (33) found that obesity rates classified by ABSI were considerably lower than those organized by BMI, which may contribute to the poor association between ABSI and DM, given the close link between obesity and DM (5). CI was the most relevant index of DM in young adults under 40. Different obesity indices may correlate with DMdifferently based on age. Young adults are less likely to develop abdominal obesity compared to middle-aged and older adults (21).

Certain obesity indices, such as WHtR, BRI, and CI, have a stronger association with DM after a certain point, possibly due to the threshold effect of obesity and insulin resistance (35). Previous studies have also shown a J-shaped risk of developing DM with certain obesity indices (15). The relationship between WC, VAI, and DM is bidirectional.Within a specific range, WC and VAI are negatively linked to DM risk, but beyond that range, they are positively related. Defining obesity based on these measures may result in the “obesity paradox.” (8). Optimal WC and VAI levels may lower DM risk, while extreme values could increase it. Nonlinear associations should be considered for targeted interventions targeting specific adiposity ranges.

All obesity indices, particularly CI, strongly correlate with early-onset DM. Studies have shown that CI is the most relevant index for cardiovascular risk in young adults compared to other indices like BMI, WC, BRI, and ABSI (35). BMI, VAI, LAP, and CMI had a nonlinear relationship with early-onset DM, but further analysis was limited due to few events and wide confidence intervals. VAI showed a bidirectional association with early-onset DM, indicating it may have a unique role in preventing and managing the condition. We searched PubMed for studies on overweight, obesity, early-onset DM, and BMI but found limited relevant research. Some studies in adolescents suggest that higher BMI is linked to a higher risk of early-onset DM (36), but there is a lack of research comparing different obesity indices. There are significantly fewer studies on early-onset DM, highlighting the need for more research and attention.

The study found that the CMI is best for predicting short-term DM risk (2–4 years), and the LAP is best for predicting medium to long-term DM risk (4–12 years) due to their comprehensive inclusion of various risk factors. These findings are consistent with prior research and offer insights into how these associations evolve over time (12, 15). LAP shows gradual metabolic deterioration related to DM risk, while CMI gives immediate information about DM risk. This means that DM risk is constantly changing. The relationships between CMI, LAP, and DM risk were consistent across different populations. This has important implications for managing DM risk based on obesity indices. The study also found the best cutoff values for each index using the Youden index. Results were close to the obesity cutoff points for each index (10–17). The study only looked at early-onset DM over 12 years due to few occurrences. WHtR was the best predictor of early-onset DM, followed by BMI and WC. This may be due to less dyslipidemia in young adults (32, 37). These findings should be confirmed in a larger and more diverse population.

Obesity is a significant factor in the development of DM, as it increases substances that lead to insulin resistance and interfere with insulin signaling pathways. Adiposity-related cytokines also trigger an inflammatory response that impairs insulin-responsive tissues, making it harder to control blood sugar levels (5). Magnetic resonance quantification is the most accurate method for measuring body fat (38), but it is not practical for general population screening due to its time-consuming and costly nature. Therefore, simpler and more cost-effective indicators like BMI are commonly used. However, non-traditional obesity indices, such as composite indices using height, weight, and waist circumference, are supported in this study. CMI and LAP were good predictors of DM risk but were not easily accessible due to the need for lipid measurements. However, new biosensor technology has made measuring these parameters more convenient (39). DM organizations globally recommend weight management and weight-loss medications for treating DM. Orlistat and glucagon-like peptide-1 receptor antagonists are widely used and have shown considerable efficacy in weight loss and glucose lowering (40, 41). However, the majority of previous studies have focused on BMI and WC. Thus, this study suggests incorporating non-traditional obesity indices in the observation of DM to gather more relevant characteristics and differences. Moreover, obesity is closely associated with DM complications, cardiovascular disease, cancer, and other illnesses (6). Hence, it is necessary to research various obesity indices in these diseases to improve decision-making abilities in clinical practice and assist disease management and prevention.

### Advantages and limitations of research

This study has strengths such as its long-term design, large sample size, and use of multiple obesity indices for analysis. It is the first to examine the relationship between obesity indices and early-onset DM risk. However, the findings may not be generalizable to other populations. Further studies with different people are needed to confirm the generalizability of our findings. Secondly, the study diagnosed DM using FPG, HbA1c levels, and self-reporting, potentially underestimating prevalence. It did not differentiate between type 1 and type 2 DM, but type 1 is rare in the Japanese population (2 per 100,000 persons) (42). Additionally, the lack of follow-up data and limited early-onset DM cases may have affected our analysis, but our study still offers valuable insights into the link between obesity and early-onset DM.

## Conclusions

In conclusion, we emphasize the significance of adiposity in the development of DM and the use of non-traditional obesity-related indices for predicting the risk of these diseases. Integrating these indices into clinical practice can help healthcare providers identify individuals at higher risk of DM and implement personalized interventions to prevent the increasing burden of obesity-related DM.

## Data Availability

The original contributions presented in the study are included in the article/**Supplementary Material**. Further inquiries can be directed to the corresponding authors.
