# Supplementary material for: Relationship between traditional and non-traditional obesity parameters and diabetes and early-onset diabetes: an analysis based on a large cohort
Source: Front Endocrinol (Lausanne). 2025 Sep 9;16:1593917. doi: 10.3389/fendo.2025.1593917 (PMC12454049; doi:10.3389/fendo.2025.1593917)
Supplement: Supplementary file 1 [file DataSheet1.docx]

**Obesity-related indices and diabetes, early-onset diabetes: insights from a large cohort study**

**Supplementary Table 1** Boundary values for each obesity index grouped

according to quartiles

|  | **Q1** | **Q2** | **Q3** | **Q4** |
| --- | --- | --- | --- | --- |
| **DM cohort, n** | 3864 | 3863 | 3863 | 3863 |
| BMI | 13.77, 19.89 | 19.89, 21.79 | 21.79, 23.92 | 23.92, 49.92 |
| WC | 36.9, 70 | 70, 76 | 76, 82.5 | 82.5, 130 |
| WHtR | 0.21, 0.43 | 0.43, 0.46 | 0.46, 0.49 | 0.49,0.79 |
| VAI | 0.049, 0.46 | 0.46, 0.74 | 0.74, 1.26 | 1.26, 21.15 |
| LAP | -17.77, 4.74 | 4.74, 9.76 | 9.76, 19.51 | 19.51, 212.58 |
| CI | 0.58, 1.12 | 1.12, 1.16 | 1.16, 1.21 | 1.21, 1.45 |
| CMI | 0.01, 0.13 | 0.13, 0.23 | 0.23, 0.44 | 0.44, 7.99 |
| AIP | -3.46, -1.20 | -1.20, -0.68 | -0.68, -0.10 | -0.10, 2.81 |
| ABSI | 0.38, 0.73 | 0.73, 0.76 | 0.76, 0.78 | 0.78, 0.96 |
| BRI | -0.45, 2.10 | 2.10, 2.64 | 2.64, 3.26 | 3.26, 10.39 |
| **Early-onset DM cohort, n** | 1396 | 1396 | 1396 | 1396 |
| BMI | 14.41, 19.46 | 19.46, 21.30 | 21.30, 23.54 | 23.54, 49.92 |
| WC | 48.5, 68 | 68, 74 | 74, 80.6 | 80.6, 130 |
| WHtR | 0.28, 0.42 | 0.42, 0.45 | 0.45, 0.48 | 0.48, 0.79 |
| VAI | 0.05, 0.39 | 0.39, 0.61 | 0.61, 1.06 | 1.06, 14.10 |
| LAP | -12.30, 3.48 | 3.48, 7.08 | 7.08, 14.90 | 14.94, 212.58 |
| CI | 0.75, 1.10 | 1.10, 1.15 | 1.15, 1.19 | 1.19, 1.44 |
| CMI | 0.01, 0.11 | 0.11, 0.19 | 0.19, 0.36 | 0.36, 6.17 |
| AIP | -3.46, -1.35 | -1.35, -0.86 | -0.86, -0.26 | -0.26, 2.46 |
| ABSI | 0.49, 0.73 | 0.73, 0.75 | 0.75, 0.77 | 0.77, 0.92 |
| BRI | 0.15, 1.93 | 1.93, 2.39 | 2.39, 2.97 | 2.97, 10.39 |

Abbreviations can be seen in **Table 1**

**Supplementary Table 2** Restricted cubic spline plot kont locations

| Number of knots | Knot locations expressed in quantiles of the x-axis variable | | | | | | |
| --- | --- | --- | --- | --- | --- | --- | --- |
| 3 | 0.1 | 0.5 | 0.9 |  |  |  |  |
| 4 | 0.05 | 0.35 | 0.65 | 0.95 |  |  |  |
| 5 | 0.05 | 0.275 | 0.5 | 0.725 | 0.95 |  |  |
| 6 | 0.05 | 0.23 | 0.41 | 0.59 | 0.77 | 0.95 |  |
| 7 | 0.025 | 0.1833 | 0.3417 | 0.5 | 0.6583 | 0.8167 | 0.975 |

**Supplementary Table 3** Correlation analysis between

obesity indices and METS-IR

| **Obesity-related indices** | **DM cohort** | | **Early-onset DM cohort** | |
| --- | --- | --- | --- | --- |
|  | r | P value | r | P value |
| BMI | 0.915 | < 0.001 | 0.927 | < 0.001 |
| WC | 0.842 | < 0.001 | 0.854 | < 0.001 |
| WHtR | 0.783 | < 0.001 | 0.791 | < 0.001 |
| VAI | 0.716 | < 0.001 | 0.708 | < 0.001 |
| LAP | 0.840 | < 0.001 | 0.838 | < 0.001 |
| CI | 0.488 | < 0.001 | 0.476 | < 0.001 |
| CMI | 0.811 | < 0.001 | 0.810 | < 0.001 |
| AIP | 0.759 | < 0.001 | 0.759 | < 0.001 |
| ABSI | 0.154 | < 0.001 | 0.084 | < 0.001 |
| BRI | 0.783 | < 0.001 | 0.791 | < 0.001 |

Abbreviations can be seen in **Table 1**

**Supplementary Table 4** PH test for each

obesity index

| **Obesity-related indices** | **DM cohort** | **Early-onset DM cohort** |
| --- | --- | --- |
| BMI | 0.637 | 0.286 |
| WC | 0.365 | 0.236 |
| WHtR | 0.664 | 0.138 |
| VAI | 0.083 | 0.918 |
| LAP | 0.864 | 0.526 |
| CI | 0.505 | 0.18 |
| CMI | 0.168 | 0.604 |
| AIP | 0.594 | 0.589 |
| ABSI | 0.726 | 0.51 |
| BRI | 0.247 | 0.169 |

PH proportional risk hypothesis,

other abbreviations can be seen in **Table 1**

**Supplementary Table 5** Collinearity test of covariables

|  | VIF | | | | | | | | | |
| --- | --- | --- | --- | --- | --- | --- | --- | --- | --- | --- |
| **DM cohort** | | | | | | | | | | |
| BMI | 1.8 | - | - | - | - | - | - | - | - | - |
| WC | - | 2 | - | - | - | - | - | - | - | - |
| WHtR | - | - | 1.7 | - | - | - | - | - | - | - |
| VAI | - | - | - | 1.8 | - | - | - | - | - | - |
| LAP | - | - | - | - | 1.9 | - | - | - | - | - |
| CI | - | - | - | - | - | 1.3 | - | - | - | - |
| CMI | - | - | - | - | - | - | 1.9 | - | - | - |
| AIP | - | - | - | - | - | - | - | 3.3 | - | - |
| ABSI | - | - | - | - | - | - | - | - | 1.1 | - |
| BRI | - | - | - | - | - | - | - | - | - | 1.7 |
| Age | 1.2 | 1.2 | 1.2 | 1.2 | 1.2 | 1.3 | 1.2 | 1.2 | 1.3 | 1.2 |
| Gender | 1.9 | 2 | 2 | 2 | 2 | 1.9 | 1.9 | 1.9 | 1.9 | 2 |
| SBP | 1.4 | 1.4 | 1.4 | 1.3 | 1.3 | 1.3 | 1.3 | 1.3 | 1.3 | 1.4 |
| FPG | 1.5 | 1.5 | 1.5 | 1.5 | 1.5 | 1.5 | 1.5 | 1.5 | 1.5 | 1.5 |
| HbA1c | 1.2 | 1.3 | 1.3 | 1.2 | 1.2 | 1.3 | 1.2 | 1.2 | 1.3 | 1.3 |
| TC | 1.3 | 1.3 | 1.3 | 1.3 | 1.4 | 1.3 | 1.3 | 1.5 | 1.2 | 1.3 |
| HDL-C | 1.5 | 1.5 | 1.6 | 2 | 1.7 | 1.5 | 1.9 | 2.8 | 1.4 | 1.5 |
| ALT | 4.1 | 4.1 | 4.1 | 4 | 4.1 | 4 | 4.1 | 4.1 | 4 | 4.1 |
| AST | 3.3 | 3.3 | 3.3 | 3.3 | 3.3 | 3.3 | 3.3 | 3.3 | 3.3 | 3.3 |
| GGT | 1.5 | 1.5 | 1.5 | 1.5 | 1.5 | 1.5 | 1.5 | 1.5 | 1.5 | 1.5 |
| Fatty liver | 1.5 | 1.5 | 1.5 | 1.4 | 1.5 | 1.4 | 1.5 | 1.4 | 1.4 | 1.5 |
| Smoking | 1.4 | 1.4 | 1.4 | 1.4 | 1.4 | 1.4 | 1.4 | 1.4 | 1.4 | 1.4 |
| Drinking | 1.3 | 1.3 | 1.3 | 1.3 | 1.3 | 1.3 | 1.3 | 1.3 | 1.3 | 1.3 |
| Exercise | 1 | 1 | 1 | 1 | 1 | 1 | 1 | 1 | 1 | 1 |
| **Early-onset DM cohort** | | | | | | | | | | |
| BMI | 1.2 | - | - | - | - | - | - | - | - | - |
| WC | - | 1.3 | - | - | - | - | - | - | - | - |
| WHtR | - | - | 1.1 | - | - | - | - | - | - | - |
| VAI | - | - | - | 1.1 | - | - | - | - | - | - |
| LAP | - | - | - | - | 1.1 | - | - | - | - | - |
| CI | - | - | - | - | - | 1.1 | - | - | - | - |
| CMI | - | - | - | - | - | - | 1.2 | - | - | - |
| AIP | - | - | - | - | - | - | - | 1.3 | - | - |
| ABSI | - | - | - | - | - | - | - | - | 1 | - |
| BRI | - | - | - | - | - | - | - | - | - | 1.1 |
| Age | 1 | 1 | 1 | 1 | 1 | 1 | 1 | 1 | 1 | 1 |
| Gender | 1.2 | 1.3 | 1.1 | 1.1 | 1.1 | 1.1 | 1.2 | 1.3 | 1 | 1 |

VIF variance inflation factor, other abbreviations can be seen in **Table 1**

**Supplementary Table 6** Cox regression analysis of obesity indices and DM and early-onset DM

|  | **HR (95% CI) *P* value** | | | |
| --- | --- | --- | --- | --- |
|  | **Unadjusted model** | **Model 1** | **Model 2** | **Model 3** |
| **DM** |  |  |  |  |
| BMI (per SD incerase) | 1.97 (1.84, 2.10) <0.001 | 2.05 (1.90, 2.22) <0.001 | 1.31 (1.17, 1.47) <0.001 | 1.37 (1.21, 1.55) <0.001 |
| WC (per SD incerase) | **2.25 (2.07, 2.44) <0.001** | **2.26 (2.06, 2.48) <0.001** | 1.37 (1.21, 1.56) <0.001 | 1.44 (1.25, 1.65) <0.001 |
| WHtR (per SD incerase) | 2.24 (2.07, 2.42) <0.001 | 2.19 (2.01, 2.39) <0.001 | **1.39 (1.24, 1.56) <0.001** | **1.47 (1.30, 1.66) <0.001** |
| VAI (per SD incerase) | 1.34 (1.30, 1.38) <0.001 | 1.31 (1.27, 1.36) <0.001 | 1.11 (1.02, 1.20) 0.010 | 1.07 (0.98, 1.18) 0.147 |
| LAP (per SD incerase) | 1.60 (1.53, 1.67) <0.001 | 1.53 (1.46, 1.61) <0.001 | 1.18 (1.09, 1.27) <0.001 | 1.18 (1.09, 1.27) <0.001 |
| CI (per SD incerase) | 2.15 (1.94, 2.38) <0.001 | 1.91 (1.70, 2.13) <0.001 | 1.28 (1.13, 1.44) <0.001 | 1.32 (1.16, 1.51) <0.001 |
| CMI (per SD incerase) | 1.36 (1.32, 1.40) <0.001 | 1.33 (1.28, 1.37) <0.001 | 1.12 (1.04, 1.21) 0.004 | 1.09 (1.00, 1.20) 0.053 |
| AIP (per SD incerase) | 2.22 (2.02, 2.45) <0.001 | 2.09 (1.87, 2.33) <0.001 | 1.13 (0.94, 1.37) 0.196 | 1.05 (0.86, 1.30) 0.618 |
| ABSI (per SD incerase) | 1.58 (1.43, 1.75) <0.001 | 1.35 (1.21, 1.51) <0.001 | 1.16 (1.03, 1.30) 0.014 | 1.18 (1.04, 1.34) 0.011 |
| BRI (per SD incerase) | 2.00 (1.88, 2.12) <0.001 | 2.00 (1.86, 2.14) <0.001 | 1.35 (1.22, 1.49) <0.001 | 1.41 (1.27, 1.57) <0.001 |
| **Early-onset DM** |  |  |  |  |
| BMI (per SD incerase) | 2.00 (1.70, 2.35) <0.001 | 2.28 (1.82, 2.84) <0.001 |  |  |
| WC (per SD incerase) | 2.44 (1.97, 3.03) <0.001 | 2.53 (1.99, 3.20) <0.001 |  |  |
| WHtR (per SD incerase) | 2.25 (1.87, 2.71) <0.001 | 2.29 (1.87, 2.82) <0.001 |  |  |
| VAI (per SD incerase) | 1.37 (1.23, 1.53) <0.001 | 1.34 (1.18, 1.51) <0.001 |  |  |
| LAP (per SD incerase) | 1.59 (1.39, 1.81) <0.001 | 1.49 (1.30, 1.72) <0.001 |  |  |
| CI (per SD incerase) | **2.74 (2.01, 3.73) <0.001** | **2.63 (1.88, 3.66) <0.001** |  |  |
| CMI (per SD incerase) | 1.43 (1.28, 1.59) <0.001 | 1.35 (1.20, 1.52) <0.001 |  |  |
| AIP (per SD incerase) | 2.17 (1.53, 3.06) <0.001 | 2.01 (1.36, 2.98) <0.001 |  |  |
| ABSI (per SD incerase) | 1.74 (1.21, 2.49) 0.003 | 1.70 (1.15, 2.52) 0.008 |  |  |
| BRI (per SD incerase) | 1.90 (1.65, 2.18) <0.001 | 1.96 (1.67, 2.29) <0.001 |  |  |

Model 1: adjusted for age and gender;

Model 2: adjusted for age, gender, SBP, FPG, HbA1c, TC, TG, HDL, ALT, AST, GGT, smoking, drinking, and exercise;

Model 3 excludes study participants with less than 2 years of follow-up based on Model 2;

HR hazard ratio, CI confidence interval, other abbreviations can be seen in **Table 1**

**Supplementary Table 7** Sectional Cox regression analysis of obesity indices and DM

| **Obesity-related indices** | **Events/**  **Participants** | HR (95%CI) ***P*** value | ***P*** for log-likelihood ratio test |
| --- | --- | --- | --- |
| **WC (per SD incerase)** |  |  | <0.001 |
| <72.1 | 38/5241 | 1.39 (0.90, 2.15) 0.135 |  |
| 72.1-78.5 | 63/4023 | 0.70 (0.53, 0.93) 0.013 |  |
| >78.5 | 272/6151 | 1.36 (1.23, 1.50) <0.001 |  |
| **WHtR (per SD incerase)** |  |  | 0.012 |
| <0.42 | 15/3134 | 0.95 (0.52, 1.75) 0.876 |  |
| 0.42-0.47 | 76/5800 | 0.81 (0.63, 1.03) 0.082 |  |
| >0.47 | 282/6159 | 1.36 (1.24, 1.50) <0.001 |  |
| **VAI (per SD incerase)** |  |  | 0.011 |
| <0.43 | 28/3405 | 0.56 (0.36, 0.90) 0.015 |  |
| ≥0.43 | 345/12048 | 1.11 (1.02, 1.20) 0.021 |  |
| **CI (per SD incerase)** |  |  | 0.003 |
| <1.10 | 22/2842 | 1.62 (0.80, 3.29) 0.185 |  |
| 1.10-1.16 | 67/4715 | 0.85 (0.65, 1.10) 0.203 |  |
| >1.16 | 284/7896 | 1.30 (1.17, 1.43) <0.001 |  |
| **BRI (per SD incerase)** |  |  | 0.022 |
| <2.04 | 20/3511 | 1.04 (0.67, 1.61) 0.870 |  |
| 2.04-2.73 | 63/4885 | 1.01 (0.76, 1.35) 0.931 |  |
| >2.73 | 290/7057 | 1.31 (1.20, 1.44) <0.001 |  |

Above models are adjusted for age, gender, SBP, FPG, HbA1c, TC, TG, HDL, ALT, AST, GGT, smoking, drinking, and exercise.

HR hazard ratio, CI confidence interval, other abbreviations can be

seen in **Table 1.**

**Supplementary Table 8** e-values of each obesity index

|  | **e-value** | |
| --- | --- | --- |
|  | **DM** | **Early-onset DM** |
| **BMI (per SD incerase)** | 1.95 | 3.99 |
| **WC (per SD incerase)** | 2.08 | 4.5 |
| **WHtR (per SD incerase)** | 2.13 | 4.01 |
| **VAI (per SD incerase)** | 1.46 | 2.01 |
| **LAP (per SD incerase)** | 1.64 | 2.34 |
| **CI (per SD incerase)** | 1.88 | 4.7 |
| **CMI (per SD incerase)** | 1.49 | 2.04 |
| **AIP (per SD incerase)** | 1.51 | 3.43 |
| **ABSI (per SD incerase)** | 1.59 | 2.79 |
| **BRI (per SD incerase)** | 2.04 | 3.33 |

Abbreviations can be seen in **Table 1**

**Supplementary Figure 9** Subgroup analysis of obesity indices and DM

|  | **HR (95% CI) *P*_interaction_** | | | | | | | | |  |
| --- | --- | --- | --- | --- | --- | --- | --- | --- | --- | --- |
|  | **BMI** | | **WC** | | **WHtR** | | **VAI** | | **LAP** | |
| **Age, years** |  | 0.131 |  | 0.218 |  | 0.177 |  | 0.607 |  | 0.220 |
| 20-39 (n = 5 584) | 1.41 (1.18, 1.69) |  | 1.49 (1.23, 1.82) |  | 1.52 (1.27, 1.82) |  | 1.15 (1.03, 1.28) |  | 1.25 (1.11, 1.41) |  |
| 40-59 (n = 9 157) | 1.27 (1.12, 1.44) |  | 1.36 (1.18, 1.57) |  | 1.39 (1.22, 1.58) |  | 1.09 (0.99, 1.20) |  | 1.17 (1.07, 1.28) |  |
| ≥60 (n = 712) | 0.88 (0.56, 1.38) |  | 1.00 (0.66, 1.52) |  | 1.03 (0.70, 1.52) |  | 1.03 (0.82, 1.30) |  | 1.00 (0.77, 1.29) |  |
| **Gender** |  | 0.476 |  | 0.313 |  | 0.526 |  | 0.820 |  | 0.094 |
| Females (n = 7 034) | 1.37 (1.15, 1.64) |  | 1.48 (1.22, 1.78) |  | 1.44 (1.23, 1.69) |  | 1.12 (0.98, 1.28) |  | 1.32 (1.15, 1.52) |  |
| Males (n = 8 419) | 1.28 (1.12, 1.46) |  | 1.32 (1.14, 1.53) |  | 1.35 (1.18, 1.56) |  | 1.10 (1.01, 1.20) |  | 1.16 (1.07, 1.26) |  |
| **Fatty liver** |  | 0.105 |  | 0.015 |  | 0.041 |  | 0.643 |  | 0.390 |
| No (n = 12 716) | 1.15 (0.94, 1.40) |  | 1.13 (0.92, 1.38) |  | 1.19 (0.98, 1.44) |  | 1.08 (0.94, 1.24) |  | 1.11 (0.94, 1.31) |  |
| Yes (n = 2 737) | 1.38 (1.21, 1.58) |  | 1.52 (1.31, 1.76) |  | 1.50 (1.31, 1.72) |  | 1.12 (1.02, 1.22) |  | 1.19 (1.10, 1.29) |  |
| **Hypertension** |  | 0.025 |  | 0.137 |  | 0.025 |  | 0.936 |  | 0.142 |
| No (n = 14 491) | 1.39 (1.23, 1.57) |  | 1.43 (1.25, 1.64) |  | 1.46 (1.30, 1.65) |  | 1.11 (1.02, 1.21) |  | 1.22 (1.12, 1.33) |  |
| Yes (n = 962) | 1.07 (0.86, 1.33) |  | 1.17 (0.91, 1.50) |  | 1.08 (0.84, 1.39) |  | 1.10 (0.93, 1.30) |  | 1.09 (0.93, 1.26) |  |
|  | **CI** | | **CMI** | | **AIP** | | **ABSI** | | **BRI** | |
| **Age, years** |  | 0.277 |  | 0.428 |  | 0.438 |  | 0.761 |  | 0.264 |
| 20-39 (n = 5 584) | 1.52 (1.17, 1.96) |  | 1.19 (1.06, 1.33) |  | 1.30 (0.98, 1.72) |  | 1.30 (0.97, 1.75) |  | 1.42 (1.23, 1.64) |  |
| 40-59 (n = 9 157) | 1.28 (1.12, 1.48) |  | 1.10 (1.01, 1.21) |  | 1.10 (0.89, 1.35) |  | 1.17 (1.02, 1.33) |  | 1.35 (1.20, 1.51) |  |
| ≥60 (n = 712) | 1.07 (0.76, 1.53) |  | 1.03 (0.82, 1.31) |  | 1.06 (0.73, 1.53) |  | 1.12 (0.80, 1.58) |  | 1.04 (0.72, 1.50) |  |
| **Gender** |  | 0.354 |  | 0.419 |  | 0.269 |  | 0.303 |  | 0.626 |
| Females (n = 7 034) | 1.36 (1.14, 1.63) |  | 1.20 (1.01, 1.42) |  | 1.29 (0.96, 1.74) |  | 1.25 (1.04, 1.49) |  | 1.38 (1.21, 1.58) |  |
| Males (n = 8 419) | 1.22 (1.05, 1.43) |  | 1.12 (1.03, 1.21) |  | 1.10 (0.91, 1.34) |  | 1.10 (0.95, 1.28) |  | 1.32 (1.17, 1.50) |  |
| **Supplementary Table 9 continued** | | | | | | | | | | |
| **Fatty liver** |  | 0.016 |  | 0.766 |  | 0.821 |  | 0.123 |  | 0.127 |
| No (n = 12 716) | 1.09 (0.91, 1.30) |  | 1.11 (0.97, 1.26) |  | 1.15 (0.91, 1.47) |  | 1.05 (0.88, 1.24) |  | 1.80 (1.55, 2.09) |  |
| Yes (n = 2 737) | 1.45 (1.24, 1.71) |  | 1.13 (1.04, 1.23) |  | 1.12 (0.91, 1.38) |  | 1.25 (1.07, 1.46) |  | 1.41 (1.26, 1.57) |  |
| **Hypertension** |  | 0.745 |  | 0.957 |  | 0.862 |  | 0.691 |  | 0.014 |
| No (n = 14 491) | 1.29 (1.14, 1.47) |  | 1.13 (1.04, 1.23) |  | 1.14 (0.94, 1.39) |  | 1.14 (1.01, 1.29) |  | 1.42 (1.28, 1.58) |  |
| Yes (n = 962) | 1.22 (0.86, 1.72) |  | 1.12 (0.97, 1.30) |  | 1.11 (0.78, 1.57) |  | 1.24 (0.86, 1.77) |  | 1.07 (0.86, 1.33) |  |

Above models are adjusted for age, gender, SBP, FPG, HbA1c, TC, TG, HDL, ALT, AST, GGT, smoking, drinking, and exercise;

Each model did not adjust for stratification variables;

HR hazard ratio, CI confidence interval, other abbreviations can be seen in **Table 1**

**Supplementary Table 10** C-index for obesity Index

predicting DM and early-onset DM

| **Obesity-related indices** | **C-index (95%CI)** | |
| --- | --- | --- |
|  | **DM cohort** | **Early-onset DM cohort** |
| BMI | 0.734 (0.705, 0.763) | 0.772 (0.680, 0.864) |
| WC | 0.750 (0.723, 0.777) | 0.771 (0.665, 0.877) |
| WHtR | 0.747 (0.720, 0.774) | 0.772 (0.664, 0.880) |
| VAI | 0.725 (0.694, 0.756) | 0.678 (0.535, 0.821) |
| LAP | 0.757 (0.730, 0.784) | 0.744 (0.628, 0.860) |
| CI | 0.708 (0.681, 0.735) | 0.728 (0.614, 0.842) |
| CMI | 0.746 (0.719, 0.773) | 0.702 (0.569, 0.835) |
| AIP | 0.728 (0.699, 0.757) | 0.677 (0.544, 0.810) |
| ABSI | 0.626 (0.597, 0.655) | 0.627 (0.515, 0.739) |
| BRI | 0.747 (0.720, 0.774) | 0.772 (0.664, 0.880) |

C-index concordance index, CI confidence interval,

other abbreviations can be seen in **Table 1**

**Supplementary Table 11** Pairwise comparison of c-index for obesity-related

indices using Hanley & McNeil test

| Variable 1 | Variable 2 | Statistics | *P-*value | Statistical method |
| --- | --- | --- | --- | --- |
| BMI | WC | -1.366 | 0.1719 | DeLong's test |
| BMI | WHtR | -1.45 | 0.1471 | DeLong's test |
| BMI | VAI | -0.57098 | 0.568 | DeLong's test |
| BMI | LAP | -3.0371 | 0.0024 | DeLong's test |
| BMI | CI | 2.8606 | 0.0042 | DeLong's test |
| BMI | CMI | -2.1121 | 0.0347 | DeLong's test |
| BMI | AIP | -0.83097 | 0.406 | DeLong's test |
| BMI | ABSI | 6.7064 | < 0.0001 | DeLong's test |
| BMI | BRI | -1.45 | 0.1471 | DeLong's test |
| WC | WHtR | 0.0079756 | 0.9936 | DeLong's test |
| WC | VAI | 0.10439 | 0.9169 | DeLong's test |
| WC | LAP | -2.1315 | 0.033 | DeLong's test |
| WC | CI | 5.9225 | < 0.0001 | DeLong's test |
| WC | CMI | -1.3513 | 0.1766 | DeLong's test |
| WC | AIP | -0.14068 | 0.8881 | DeLong's test |
| WC | ABSI | 9.7293 | < 0.0001 | DeLong's test |
| WC | BRI | 0.0079756 | 0.9936 | DeLong's test |
| WHtR | VAI | 0.10009 | 0.9203 | DeLong's test |
| WHtR | LAP | -2.047 | 0.0407 | DeLong's test |
| WHtR | CI | 5.4559 | < 0.0001 | DeLong's test |
| WHtR | CMI | -1.2982 | 0.1942 | DeLong's test |
| WHtR | AIP | -0.13722 | 0.8909 | DeLong's test |
| WHtR | ABSI | 9.4578 | < 0.0001 | DeLong's test |
| WHtR | BRI | 0 | 1 | DeLong's test |
| VAI | LAP | -2.6572 | 0.0079 | DeLong's test |
| VAI | CI | 2.8811 | 0.004 | DeLong's test |
| VAI | CMI | -5.8868 | < 0.0001 | DeLong's test |
| VAI | AIP | -1.1084 | 0.2677 | DeLong's test |
| VAI | ABSI | 7.2244 | < 0.0001 | DeLong's test |
| VAI | BRI | -0.10009 | 0.9203 | DeLong's test |
| LAP | CI | 5.5924 | < 0.0001 | DeLong's test |
| LAP | CMI | 0.20866 | 0.8347 | DeLong's test |
| LAP | AIP | 2.0501 | 0.0404 | DeLong's test |
| LAP | ABSI | 9.6799 | < 0.0001 | DeLong's test |
| LAP | BRI | 2.047 | 0.0407 | DeLong's test |
| CI | CMI | -4.3722 | < 0.0001 | DeLong's test |
| CI | AIP | -3.1125 | 0.0019 | DeLong's test |
| CI | ABSI | 12.671 | < 0.0001 | DeLong's test |
| CI | BRI | -5.4559 | < 0.0001 | DeLong's test |
| CMI | AIP | 8.2488 | < 0.0001 | DeLong's test |
| CMI | ABSI | 8.6701 | < 0.0001 | DeLong's test |
| CMI | BRI | 1.2982 | 0.1942 | DeLong's test |
| AIP | ABSI | 7.4381 | < 0.0001 | DeLong's test |
| AIP | BRI | 0.13722 | 0.8909 | DeLong's test |
| ABSI | BRI | -9.4578 | < 0.0001 | DeLong's test |

Abbreviations can be seen in **Table 1.**
